# Allergic sensitization to Mal d 1 without detectable specific serum IgE

**DOI:** 10.1111/pai.13891

**Published:** 2022-12-07

**Authors:** Alla Litovkina, Maria Byazrova, Evgenii Smolnikov, Alexandra Nikonova, Olga Elisyutina, Elena Fedenko, Nataliya Ilina, Oluwatoyin Akinfenwa, Raffaela Campana, Dmitry Kudlay, Rudolf Valenta, Musa Khaitov

**Affiliations:** ^1^ National Research Center Institute of Immunology FMBA of Russia Moscow Russian Federation; ^2^ Peoples' Friendship University of Russia (RUDN University) Moscow Russian Federation; ^3^ Lomonosov Moscow State University Moscow Russian Federation; ^4^ Division of Immunopathology, Department of Pathophysiology and Allergy Research, Center for Pathophysiology, Infectiology and Immunology Medical University of Vienna Vienna Austria; ^5^ Department of Clinical Immunology and Allergy Sechenov First Moscow State Medical University Moscow Russian Federation; ^6^ Karl Landsteiner University of Health Sciences Krems Austria; ^7^ Pirogov Russian National Research Medical University Moscow Russian Federation

To the Editor,

In clinical practice it is sometimes observed that allergic patients exhibit allergic symptoms or positive skin prick test results in the absence of detectable allergen‐specific IgE in serum. One example for such a condition is local allergic rhinitis (LAR) where it is hypothesized that IgE is produced only locally but does not reach the systemic circulation.^1^ In this study, 25 subjects, age from 13 to 59 years [Mean 32.6 ± 13.6], 16 males and nine females, with birch pollen‐related allergic rhinoconjunctivitis (ARC) with or without allergic asthma (AA), with (group 1: *n* = 13) or without cross‐reactive food allergy to apple (group 2: *n* = 12) (oral allergy syndrome, OAS) were enrolled (Table S1) and investigated at three time points (i.e., before the birch pollen season‐time point 1, shortly after the birch pollen season‐time point 2 and thereafter in autumn‐time point 3) (Figure 1). The diagnosis of birch pollen allergy and OAS, demographic, serological and clinical features of the patients are described in the Supplemental materials (Table S1). Two patients from group 1 and group 2, (patients 1 and 2) stood out. They contained low but clearly positive Bet v 1‐specific IgE levels before the birch pollen season (time point 1, patient 1: 0.16 kUA/L; patient 2: 0.11 kUA/L), showed increases of Bet v 1‐specific IgE shortly after the birch pollen season (time point 2, patient 1: 0.75 kUA/L, patient 2: 0.53 kUA/L), which then declined again until September at time point 3 (patient 1: 0.53 kUA/L; patient 2: 0.43 kUA/L) (Tables S2 and S3, Figures 2 and 3). Interestingly, Mal d 1‐specific IgE could not be detected in the sera obtained from patients 1 and 2 at any of the three time points even by sensitive and quantitative ImmunoCAP technology (Table S2; Figures 2 and 3).

**FIGURE 1:**
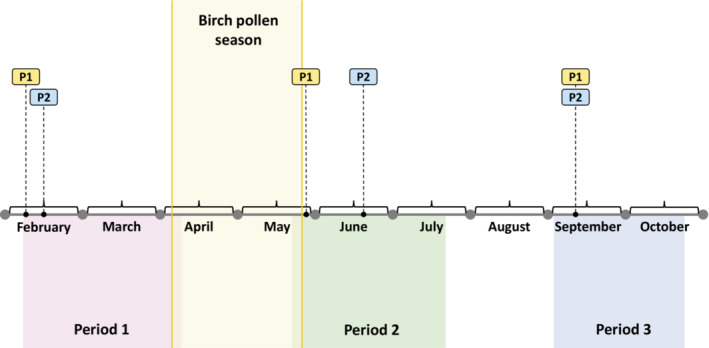
Schematic representation of experimental schedule. Flags P1 (point 1) and P2 (point 2) represent blood collection dates for patients before (period 1) and shortly after the birch pollen season (period 2) and in September (period 3). The birch pollen season was determined according to pollen monitoring data.

**FIGURE 2:**
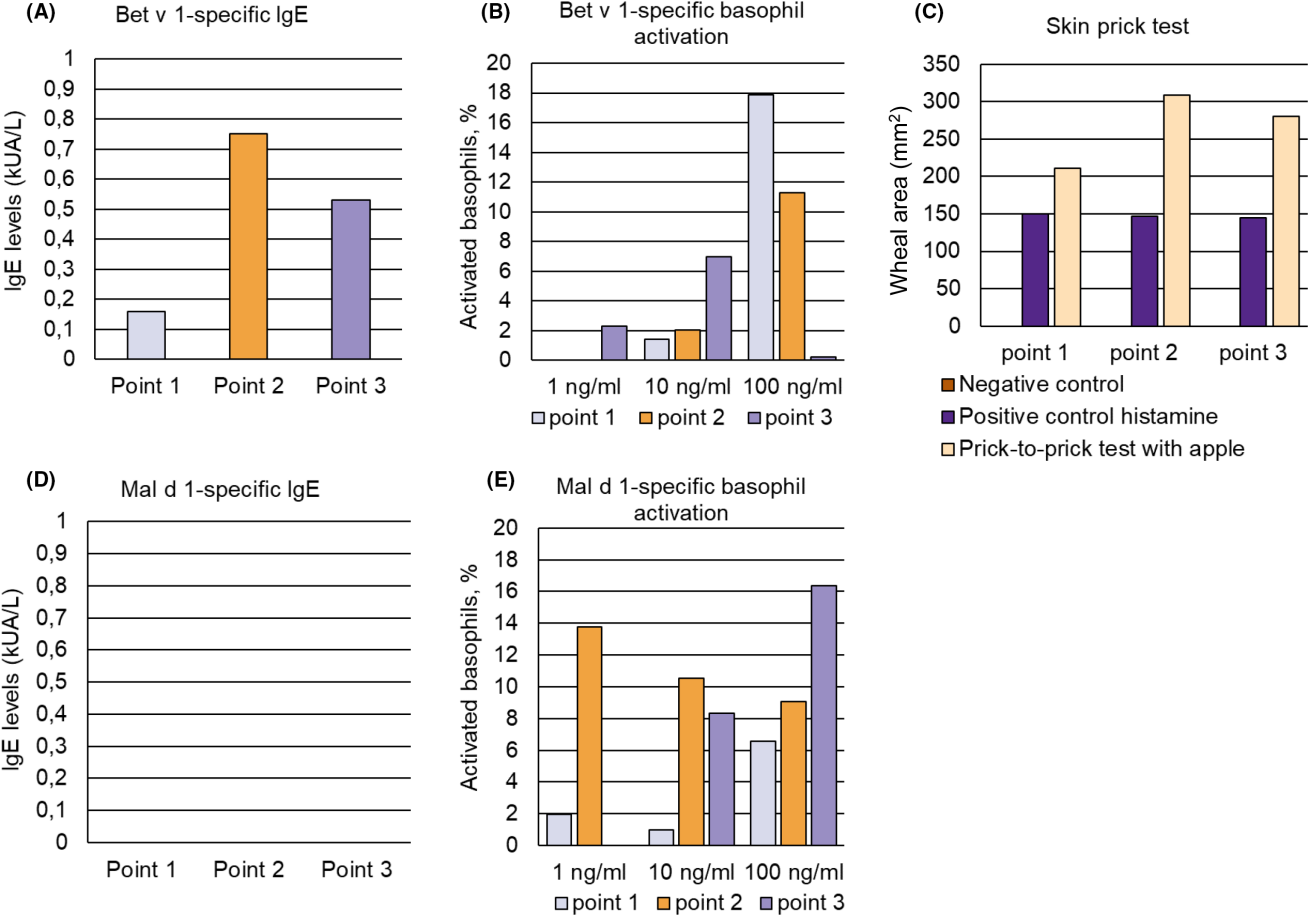
(A) Bet v 1‐specific IgE levels (y‐axis: kUA/L), (B) Bet v 1‐specific basophil activation (y‐axis: percentages of activated basophils) in response to three different concentrations of Bet v 1 (x‐axis) and (C) skin sensitivity to buffer (negative control), histamine (positive control) and apple. (D) Mal d 1‐specific IgE levels (y‐axis: kUA/L) at the three time points, (E) Mal d 1‐specific basophil activation (y‐axis: percentages of activated basophils) in response to three different concentrations of Mal d 1 (x‐axis)  determined at three different time points (Figure 1) for patient 1.

**FIGURE 3:**
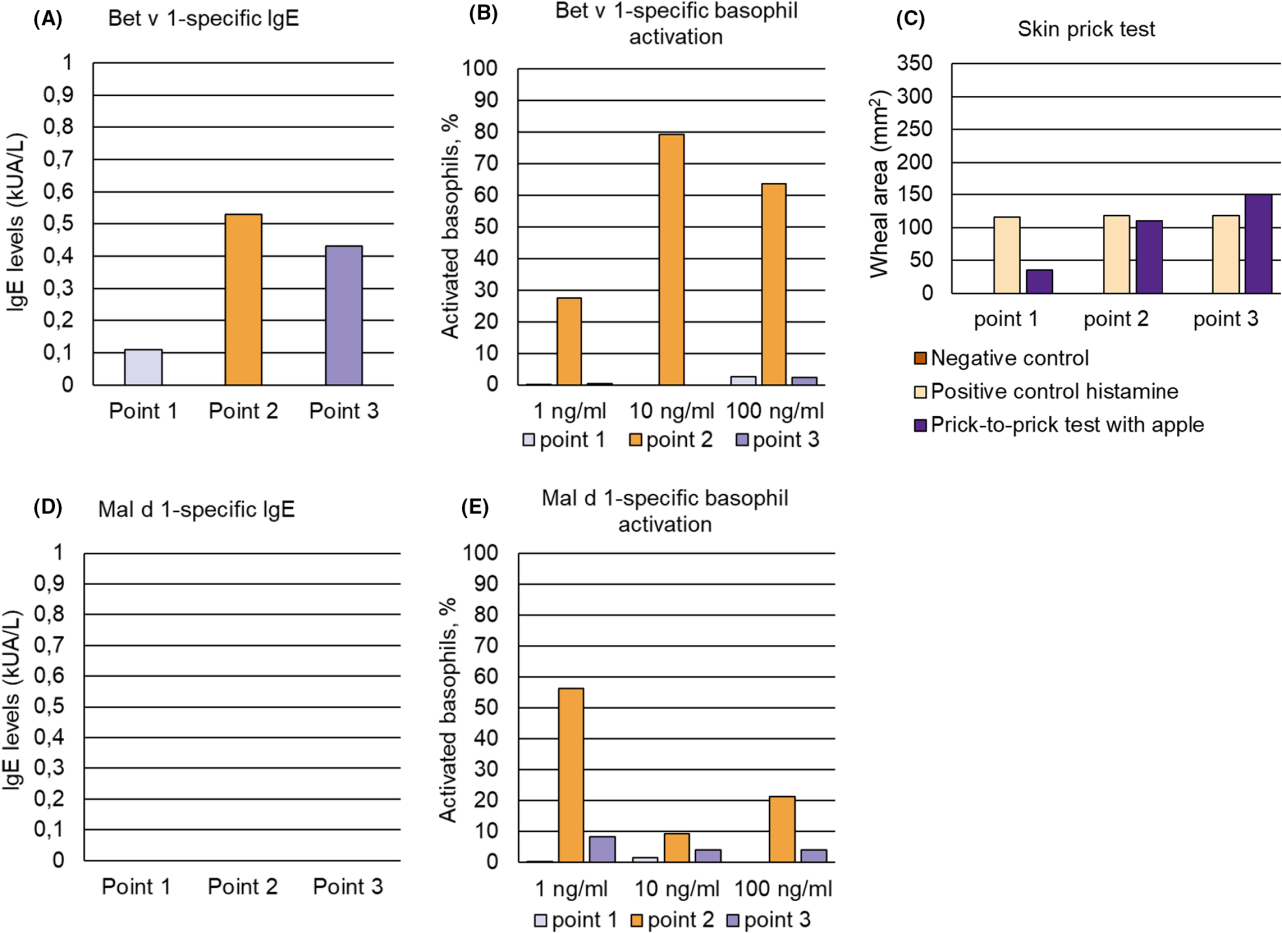
(A) Bet v 1‐specific IgE levels (y‐axis: kUA/L), (B) Bet v 1‐specific basophil activation (y‐axis: percentages of activated basophils) in response to three different concentrations of Bet v 1 (x‐axis), (C) skin sensitivity to buffer (negative control), histamine (positive control) and apple determined at three different time points (Figure 1) for patient 2, (D) Mal d 1‐specific IgE levels (y‐axis: kUA/L) and (E) Mal d 1‐specific basophil activation (y‐axis: percentages of activated basophils) in response to three different concentrations of Mal d 1 (x‐axis) at the three time points.

The two patients were young females who suffered from birch pollen‐related allergic rhinoconjunctivitis and one (patient 1) suffered also from birch pollen‐induced asthma (Table S4). Importantly, one patient suffered from OAS upon consumption of apples, especially after the birch pollen season and was positive in open food challenge with apple (Table S4). The two patients were sensitized only to few other allergens besides Bet v 1 and Mal d 1 and sensitization to other cross‐reactive food allergens (e.g., profilin, lipid transfer proteins) could be excluded (Table S5).

An increase of Bet v 1‐specific IgE levels was observed in both patients 1 and 2 at time point 2 shortly after the birch pollen season (Figures 2 and 3, Table S2), which was associated with a strong increase in Bet v 1‐specific basophil sensitivity in patient 2 (Figure 3, Table S6). The Bet v 1‐specific basophil sensitivity in patient 1 at time points 1 and 2 was comparable whereas at time point 3 patient 1 showed basophil activation with a tenfold lower Bet v 1 concentration indicating increased Bet v 1‐specific basophil sensitivity at time point 3 (Figure 2, Table S6). Exposure of basophils to anti‐IgE antibodies indicated strongly reduced IgE‐mediated basophil sensitivity in patient 1 at time point 3 as compared to time points 1 and 2 (Table S6). However, anti‐IgE‐induced basophil activation cannot be compared with allergen‐induced basophil activation because of the different molarity of the cross‐linkers and the different modes of cross‐linking (i.e., cross‐linking of IgE constant regions versus different IgE epitopes on allergens). Importantly, anti‐IgE will induce not only cross‐linking of Bet v 1 and/or Mal d 1‐specific IgE also but of IgE antibodies with different other specificities.

We could not detect at any of the three time points Mal d 1‐specific IgE in the sera of patients 1 and 2 (Table S2, Figures 2 and 3) but we found a strongly increased Mal d 1‐specific basophil sensitivity in both patients at time points 2 and 3 (Figures 2 and 3, Table S6) after the birch pollen season indicating increases of Mal d 1‐specific IgE on the patients basophils after birch pollen exposure. At time point 2 it appeared that Mal d 1 activated more basophils than Bet v 1 although the latter is the genuinely sensitizing allergen. This may be explained by the interplay of IgE antibodies with different affinities, different numbers of IgE epitopes and epitope proximity.^2^, ^3^, ^4^

The increase of Mal d 1‐specific basophil sensitivity was associated with marked increased apple‐induced skin responses at time points 2 and 3 after the birch pollen season as compared to time point 1 before the birch pollen season (Figures 2 and 3, Table S7). By contrast, skin responses to histamine were comparable at all three time points in patients 1 and 2 (Table S7). It is of note that the cumulative allergen‐specific IgE levels calculated according to ImmunoCAP ISAC results in patient 1 and patients 2 were the lowest (i.e., patient 1: 11.08 ISU; patient 2: 12.49 ISU) among all patients analyzed (Table S8) because it may explain the high activity of the low levels of cell‐bound Mal d 1‐specific IgE.

Using purified allergen molecules, our study has demonstrated that basophils and mast cells of allergic patients contain already allergen‐specific IgE before it can be detected in serum in the form of free‐allergen‐specific antibodies. This finding is different from LAR regarding the underlying mechanism because it is not a result of local IgE production without spill‐over into the systemic circulation. Furthermore, it is important because it demonstrates that allergen‐specific IgE sensitization may already occur before allergen‐specific IgE can be detected as free IgE in serum because newly produced IgE may be sequentially adsorbed by IgE receptors on cells (Figure S1). The lack of detectable Mal d 1‐specific IgE reactivity in serum despite Mal d 1‐specific basophil activation and skin reactivity may be explained by low Mal d 1‐specific IgE levels but eventually also by low avidity of Mal d 1‐specific IgE. Our results thus indicate that the early detection of allergen‐specific IgE sensitization may require basophil activation, skin testing and/or provocation testing with defined allergen molecules to detect IgE bound to cellular IgE receptors before it can be traced in serum (Figure S1).

We have studied IgE levels and allergen‐specific and IgE‐mediated effector cell activation using Mal d 1 that cross‐reacts with the major birch pollen allergen Bet v 1. This model is representative for the most common allergens, which cross‐react to a variable extent with homologous proteins from other allergen sources and isoallergens from the same allergen source.

Our results are supported by an earlier study, which has demonstrated that after administration of omalizumab, a monoclonal anti‐IgE antibody, which prevents allergen‐specific IgE from binding to the high and low affinity IgE receptor on cells, IgE sensitizations to allergen sources became detectable in serum, which were undetectable before administration of omalizumab.^5^ This effect would be explained by the fact that omalizumab blocked the binding of low levels of IgE specific for the allergen sources to cellular IgE receptors so that they became measurable in serum, which is in agreement with the notion that application of omalizumab generally increases levels of allergen‐specific free IgE in serum.

Our results are important for diagnosis of IgE‐mediated allergic sensitization in general because they reveal that a certain threshold of allergen‐specific IgE levels must be reached, which exceeds the capacity of cellular IgE receptors in the body to bind IgE before it can become detectable as free IgE in serum. In this context, it has to be borne in mind that the expression of cellular IgE receptors is regulated by the IgE levels^6^ in the blood and, therefore, together with initial increases of IgE also the expression of IgE receptors will be upregulated until a certain threshold is reached. All this must be taken into consideration when patients suffer from allergic symptoms but IgE specific for the corresponding allergens cannot be detected. In such cases basophil activation or skin testing with defined allergen molecules should be considered.

Our findings are also important when it comes to the investigation of the inception (i.e., time window of sensitization) of allergic sensitization in children because they indicate that early allergen‐specific IgE sensitization may not be accurately detected by serology. This may apply for subjects with low allergen‐specific IgE levels who are sensitized only to few allergen molecules. In fact, monitoring of allergen‐specific IgE to multiple allergen molecules in birth cohorts indeed showed that children are often sensitized only to few dominant allergen molecules in the beginning of sensitization.^7^, ^8^, ^9^ This may have important consequences for strategies for allergen‐specific prevention because it may affect the definition of primary versus secondary prevention depending on the accurate definition at what time point in life allergic sensitization has indeed occurred. Finally, our data may explain why certain patients show allergic symptoms and/or allergen‐specific positive challenge test (e.g., skin test) and basophil activation results although no specific IgE can be detected in serum. It is a limitation of our study that we have investigated only 25 birch pollen allergic patients but among those approximately 10% of patients showed a discrepancy between detection of specific IgE by tests based on effector cell activation versus IgE serology. Larger studies will be needed to determine the percentages of patients who present only effector cell‐bound‐specific IgE without detectable specific IgE in serum. One may envisage that such studies are performed as prospective longitudinal birth cohort studies in which the evolution of allergen‐specific IgE reactivity, skin test reactivity and basophil activation is studied with defined purified recombinant allergens.^10^, ^11^ However, we are currently running out regarding high quality allergen preparations for skin testing^12^ and it will, therefore, be necessary to obtain purified allergen molecules produced under Good Manufacture Practice (GMP) conditions for such trials.

The strength of our study is that it was conducted with highly purified allergen molecules and hence delivered unambiguous results because they demonstrate the presence of allergen‐specific IgE on basophils and in the tissues of allergic patients without detectable allergen‐specific IgE in serum.

## AUTHOR CONTRIBUTIONS

RV, AL, AN, MK, DK, OE designed the research studies. AL, ES, OE, EF, NI performed clinical work. AL, MB, ES, AN, OA, RC performed experiments. RV, AL, MB, ES analyzed and interpreted the data. AL wrote the manuscript with contributions from RV and ES.

## CONFLICTS OF INTEREST

Rudolf Valenta has received research grants from Viravaxx AG, Vienna, Austria, HVD Biotech, Vienna, Austria and Worg Pharmaceuticals, Hangzhou, China. He serves as a consultant for Viravaxx and Worg. The other authors have not conflicts of interest to declare.

### PEER REVIEW

The peer review history for this article is available at https://publons.com/publon/10.1111/pai.13891.

## Supporting information


Appendix S1

FigureS1

## References

[1] Prieto A , Rondón C , Eguiluz‐Gracia I , et al. Systematic evaluation of allergic phenotypes of rhinitis in children and adolescents. Pediatr Allergy Immunol. 2021;32(5):953‐962. doi:10.1111/pai.13474 33598969

[2] Christensen LH , Holm J , Lund G , Riise E , Lund K . Several distinct properties of the IgE repertoire determine effector cell degranulation in response to allergen challenge. J Allergy Clin Immunol. 2008;122(2):298‐304. doi:10.1016/j.jaci.2008.05.026 18572230

[3] Gieras A , Focke‐Tejkl M , Ball T , et al. Molecular determinants of allergen‐induced effector cell degranulation. J Allergy Clin Immunol. 2007;119(2):384‐390. doi:10.1016/j.jaci.2006.09.034 17291855

[4] Gieras A , Linhart B , Roux KH , et al. IgE epitope proximity determines immune complex shape and effector cell activation capacity. J Allergy Clin Immunol. 2016;137(5):1557‐1565. doi:10.1016/j.jaci.2015.08.055 26684291 PMC4890651

[5] Mizuma H , Tanaka A , Uchida Y , et al. Influence of omalizumab on allergen‐specific IgE in patients with adult asthma. Int Arch Allergy Immunol. 2015;168(3):165‐172. doi:10.1159/000442668 [Published correction appears in int arch allergy Immunol. 2015;168(3):218. Matsukara, Satoshi [Corrected to Matsukura, Satoshi]]. 26790100

[6] MacGlashan DW Jr , Bochner BS , Adelman DC , et al. Down‐regulation of fc(epsilon)RI expression on human basophils during in vivo treatment of atopic patients with anti‐IgE antibody. J Immunol. 1997;158(3):1438‐1445. 9013989

[7] Westman M , Lupinek C , Bousquet J , et al. Early childhood IgE reactivity to pathogenesis‐related class 10 proteins predicts allergic rhinitis in adolescence. J Allergy Clin Immunol. 2015;135(5):1199‐1206.e1‐11. doi:10.1016/j.jaci.2014.10.042 25528361 PMC6597345

[8] Hatzler L , Panetta V , Lau S , et al. Molecular spreading and predictive value of preclinical IgE response to Phleum pratense in children with hay fever. J Allergy Clin Immunol. 2012;130(4):894‐901.e5. doi:10.1016/j.jaci.2012.05.053 22841010

[9] Posa D , Perna S , Resch Y , et al. Evolution and predictive value of IgE responses toward a comprehensive panel of house dust mite allergens during the first 2 decades of life. J Allergy Clin Immunol. 2017;139(2):541‐549.e8. doi:10.1016/j.jaci.2016.08.014 27793411

[10] Purohit A , Laffer S , Metz‐Favre C , et al. Poor association between allergen‐specific serum immunoglobulin E levels, skin sensitivity and basophil degranulation: a study with recombinant birch pollen allergen bet v 1 and an immunoglobulin E detection system measuring immunoglobulin E capable of binding to fc epsilon RI. Clin Exp Allergy. 2005;35(2):186‐192. doi:10.1111/j.1365-2222.2005.02156.x 15725190

[11] Metz‐Favre C , Linhart B , Focke‐Tejkl M , et al. Skin test diagnosis of grass pollen allergy with a recombinant hybrid molecule. J Allergy Clin Immunol. 2007;120(2):315‐321. doi:10.1016/j.jaci.2007.03.046 17512042

[12] Klimek L , Hoffmann HJ , Kalpaklioglu AF , et al. In‐vivo diagnostic test allergens in Europe: a call to action and proposal for recovery plan‐an EAACI position paper. Allergy. 2020;75(9):2161‐2169. doi:10.1111/all.14329 32306414

