# Supplementary material for: Allergic sensitization to Mal d 1 without detectable specific serum IgE
Source: Pediatr Allergy Immunol. 2022 Dec 7;33(12):e13891. doi: 10.1111/pai.13891 (PMC10107674; doi:10.1111/pai.13891)
Supplement: Supplementary file 1 — Appendix S1 [file PAI-33-0-s001.docx]

**Supplementary materials:**

**Allergic sensitization to Mal d 1 without detectable specific serum IgE**

Alla O. Litovkina ^1,2^ ^§^ , Maria G. Byazrova  ^1,2,3 §^ , Evgenii V. Smolnikov ^1,2^, Alexandra A. Nikonova^1,2^, Olga G. Elisyutina^1,2^, Elena S. Fedenko^1^, Nataliya I. Ilina^1^, Oluwatoyin Akinfenwa^4^, Raffaela Campana^4^, Dmitry A. Kudlay^1,5^, Rudolf Valenta^1,4, 5, 6, ¶^ , Musa R. Khaitov^1,7^ ^¶^ .

^1^ National Research Center – Institute of Immunology FMBA of Russia, Moscow, Russian Federation

^2^ Peoples’ Friendship University of Russia (RUDN University), Moscow, Russian Federation

^3^ Lomonosov Moscow State University, Moscow, Russian Federation

^4^ Division of Immunopathology, Department of Pathophysiology and Allergy Research, Center for Pathophysiology, Infectiology and Immunology, Medical University of Vienna, Vienna, Austria

^5^ Department of Clinical Immunology and Allergy, Sechenov First Moscow State Medical University, Moscow, Russian Federation

^6^ Karl Landsteiner University of Health Sciences, Krems, Austria

^7^ Pirogov Russian National Research Medical University, Moscow, Russian Federation

^§^Contributed equally as first authors

^¶^Contributed equally as senior authors

^*^Address correspondence to:

Rudolf Valenta, MD, Division of Immunopathology, Department of Pathophysiology and Allergy Research, Center for Pathophysiology, Infectiology and Immunology, Medical University of Vienna, Vienna, Austria Email: [rudolf.valenta@meduniwien.ac.at](mailto:rudolf.valenta@meduniwien.ac.at)

**ABBREVIATIONS**

AA, allergic asthma

AB, antibody

ARC, allergic rhinoconjunctivitis

BAT, basophil activation test

BPS, birch pollination season

EDTA, Ethylenediaminetetraacetic acid

FITC, Fluorescein isothiocyanate

OAS, oral allergy syndrome

PBMC, peripheral blood mononuclear cell

PBS, Phosphate-buffered saline

SPT, skin prick tests

SPPT, skin prick-to-prick test

FACS, fluorescence-activated cell sorting

s, second

min, minute

**SUPPLEMENTARY METHODS**

**Study population, characterization of patients and study design**

# Investigated subjects were recruited from outpatient department of NRC Institute of Immunology FMBA of Russia. Twenty five subjects, from 13 to 59 years [Me = 32,6; ±σ=13,6], 16 males and 9 females, with allergic rhinoconjunctivitis (ARC) with or without allergic asthma (AA), with or without cross-reactive food allergy to apple (oral allergy syndrome, OAS) were enrolled in the study. The diagnosis of birch pollen and apple-related symptoms was based on internationally accepted criteria and included a clinical history in combination with a positive skin prick test (SPT) with birch pollen extract and the demonstration of sIgE for major birch pollen allergen Bet v 1. All patients were free of medication and specific immunotherapy during the study. Patients filled out the “International Study of Asthma and Allergies in Childhood (ISAAC)” questionnaire^E1^ which had been translated into Russian. Asthma was defined by a positive answer to either “Have you ever had wheezing or whistling in the chest at any time in the last 12 months?” or “In the last 12 months, did you have a dry cough at night, apart from a cough associated with a cold or chest infection?.”

Allergic rhinitis was defined by a positive answer to “Have you ever had any of the following symptoms for at least one hour on most days (or on most days during the season if your symptoms are seasonal): watery, runny nose, sneezing (especially severe or even bouts of sneezing), nasal obstruction, nasal itching, conjunctivits (red, itchy eyes), postnasal drip?” or “Have you ever had allergic rhinitis?”

Atopic dermatitis was defined by a positive response to “Have you ever had atopic dermatitis?” or “Did you ever have an itchy rash that was coming and going for at least six months?”

Oral allergy syndrome was assessed by questions regarding pruritus of the lips, tongue, oral mucosa, burning sensations of the tongue, swelling of the lips or the tongue, swelling of the oral mucosa, laryngeal swelling, inflammation of the tongue or of the oral mucosa, perioral skin symptoms, wheezing, dyspnoea, nausea/vomiting and gastrointestinal disorders that are associated with ingestion of apple, peach, carrot, nuts or other fruits and vegetables. The diagnosis of asthma, rhinitis, dermatitis and OAS followed the following guidelines^E2-E6^.

Allergen sensitization profiles were established by ImmunoCAP ISAC measurement (Thermofisher, Uppsala, Sweden) to more than 100 allergen molecules. According to the presence of OAS symptoms, 2 groups were formed: Group 1 included thirteen patients with ARC/AA and OAS to apple. Group 2 included twelve patients with ARC/AA without OAS to apple. There was no significant difference between the two groups regarding age and gender distribution (Supplemental Table E1). The study was approved by the Ethical Committee of the NRC Institute of immunology FMBA of Russia. Each person gave written informed consent. Then patients were clinically assessed, including a detailed case history and physical examination. Blood samples were obtained from each patient: Aliquots of 1.6 ml of blood were collected in S Monovette® (Sarstedt AG&Co, Germany) K3 EDTA 1.6 ml tubes and used for basophil activation test immediately; 4.5 ml of blood were collected in S Monovette® (Sarstedt AG&Co, Germany) neutral 4.5 ml tubes, centrifugated at 2000xg for 10 minutes in a cooled centrifuge, and the serum was transferred into plastic tubes, and anonymized serum samples were stored at -20°C until analysis. All the measurements were made at 3 time points: before the birch pollination season (BPS), 4 and 16 weeks after BPS.

Skin prick tests (SPT) with birch pollen extract (*Betula verrucosa* extract; ALK-Abello, USA) and skin prick-to prick tests (SPPT) with apple were performed according to^E7^. SPT with birch pollen extract was performed once, before the enrollment into the study, to confirm skin reactivity to birch pollen.

**Measurement and quantification of allergen-specific IgE**

Specific IgE to major birch pollen allergen Bet v 1 and to major apple allergen Mal d 1 were measured using ImmunoCAP (Phadia AB, Thermo Fisher, Sweden) according to the manufacturer’s instructions in 3 time-points: before the BPS, 4 weeks after the BPS and 16 weeks after BPS. Values of sIgE ˂0.1 kUA/L were considered negative. The IgE reactivity profile to a comprehensive set of micro-arrayed allergen molecules was performed by ImmunoCAP ISAC (Thermofisher) measurements as described^E8^. Cumulative allergen-specific IgE levels, also termed total IgE levels,were calculated for each patient by determination of the sum of allergen-specific IgE levels to each of the allergens on the chip.

**Expression and purification of recombinant Bet v 1 and Mal d 1**

The synthetic genes for recombinant Bet v 1 (GenBank accession number: [CAA33887](http://www.ncbi.nlm.nih.gov/nuccore/CAA33887)) and Mal d 1 (GenBank accession number [CAA58646](http://www.ncbi.nlm.nih.gov/nuccore/CAA58646)) were cloned in the NdeI/ EcoRI site of the expression vector pET-17b and expressed as hexa-histidine-tagged proteins in *E*. *coli* BL21-Gold (DE3) competent cells (Agilent Technologies, Cedar Creek, Texas) as described ^E9^. Cells were lysed in buffer containing 50Mm NaH_2_PO_4_, 300mM NaCl, 10mM Imidazole, pH8 using three freeze-thaw cycles followed by centrifugation at 18.900 x g for 20 minutes at 4^0^C. Thereafter, rBet v 1 and rMal d 1 were purified by nickel affinity chromatography under native conditions (Qiagen, Hilden, Germany) in buffer containing 50Mm NaH_2_PO_4_, 300mM NaCl, 250mM Imidazole, pH8. Fractions containing recombinant proteins of more than 90% purity were pooled and dialyzed against 50Mm NaH_2_PO_4_, 300mM NaCl pH 8 at 4^o^C. The purified proteins were characterized by SDS-PAGE, mass spectroscopy and circular dichroism^E8^.

**Basophil activation testing (BAT) with recombinant allergen molecules**

Percentage of the activated basophils (AB) in whole blood samples was assessed using the Allergenicity Kit (Beckman Coulter, Fullerton, CA, USA) according to the manufacturer protocol. Blood samples were collected in S Monovette® (Sarstedt AG&Co, Germany) K3 EDTA 1.6 ml tubes; after that, 800 µL of blood were used for BAT test. BAT tests were performed with purified recombinant allergens Bet v 1 and Mal d 1 in three concentrations of each allergen: 1, 10 and 100 ng/mL. The Allergenicity Positive Сontrol containing anti-IgE antibodies (0.01 mg/mL) and negative control (PBS) were run for every patient. Flow cytometry was performed using a BD FACS Canto II (BD Biosciences, USA). Single fluorochrome stained BD TM CompBeads Compensation Particles (BD Biosciences, USA) were used to set-up the machine. Compensation was set in BD FACS Diva Software (BD Biosciences, USA). Thirty thousand PBMC were acquired compensated. The results were analyzed with FlowJo™ software version v10.6.2 (Tree Star). Results are reported as the percentage of AB, identified as CRTH2pos CD203cpos CD3neg. The isotype controls (Rat IgG2a kappa Isotype Control (eBR2a), FITC, eBioscience™, Mouse IgG_1_, (PE-Cy7), MG112, (Thermofisher) and negative control (PBS) were used for gating of AB.

**Skin prick testing with apple and open oral food challenge with apple**

For SPPT we obtained material from one batch Golden Delicious apple including pulp and skin. The material was divided into equal samples, frozen and stored at -20^o^C. Before the SPPT each sample was defrosted at room temperature and used once for each patient. Histamine dihydrochloride 0.1% solution was used as a positive control. Sodium chloride 0.9% solution was used as a negative control. The results were assessed after 15 minutes and considered positive only when the wheal reaction exceeded 3 mm^2^. For SPPT the borderlines of wheals were circled using surgical skin marker (Tondaus surgical skin marker 0.5 mm, Сhina) and transferred to the transparent plastic film. Square of plotting paper (ProMEGA Ingeneer, Russia) was used as a graphic calibrator. The films were scanned using multi-function printer (HP LaserJet Pro M28w, Hewlett-Packard, CA, USA) and converted into graphic files (JPEG). The images were analyzed using ImageJ program (National Institute of Health, USA) and the results were presented as an average square of the wheal in mm^2^.

Oral provocation test (OPT) was carried out with one batch of Golden Delicious apple to avoid variation of factors affecting allergen contents ^E10, E11^ Samples of 10 g including pulp and skin were obtained by punching and immediately frozen and stored at -80^o^C. Shortly before the OPT the samples were defrosted at room temperature. The patients were given three increasing doses of apple (10 g, 30 g, 60 g) , which they chewed for 60 s. The next dose was given every 10 min if there were no symptoms of OAS. The challenge was stopped at the lowest dose when relevant subjective symptoms (itching of lips, tongue, throat, tingling, loss of sensation) were reported or objective symptoms (swelling of lips, tongue, hoarseness, perioral urticaria) were observed, or the maximum test dose of 60 g was tolerated. The results were considered positive if at least two of subjective or one objective symptom were recorded.

**SUPPLEMENTARY TABLES**

**Table E1. Demographic and clinical characterization of the patients enrolled in the study.**

|  | Group 1  (Bet v 1 allergic patients with OAS to apple), n=13 | Group 2  (Bet v 1 allergic patients without OAS to apple), n=12 |
| --- | --- | --- |
| Age, years M±SD | 29.15±12.44 | 36.33±14.9 |
| Gender (male/female) | 9/4 | 7/5 |
| Birch associated symptoms, n=25 | | |
| Asthma (AS) (total) | 6 | 4 |
| Allergic rhinitis (AR) (total) | 13 | 12 |
| Oral allergy syndrome (OAS) (total) | 13 | 0 |
| Atopic dermatitis (AD)  (total) | 5 | 3 |
| Manifestations of allergy, n | | |
| 1 type of manifestations | | |
| AR alone | 0 | 6 |
| 2 types of manifestations | | |
| AR+AS | 0 | 3 |
| AR+AD | 0 | 2 |
| AR+OAS | 3 | 0 |
| 3 types of manifestations | | |
| AR+AS+AD | 0 | 1 |
| AR+AS +OAS | 5 | 0 |
| AR+AD+OAS | 4 | 0 |
| 4 types of manifestations | | |
| AR+AS+AD+OAS | 1 | 0 |

**TABLE E2.** Bet v 1- and Mal d 1-specific IgE levels in patients 1 and 2.

|  | **rBet v 1** | | | **rMal d 1** | | |
| --- | --- | --- | --- | --- | --- | --- |
|  | **Time points** | | | **Time points** | | |
|  | 1 | 2 | 3 | 1 | 2 | 3 |
| **Patient 1** | 0.16 | 0.75 | 0.53 | 0 | 0 | 0 |
| **Patient 2** | 0.11 | 0.53 | 0.43 | 0 | 0 | 0 |

**TABLE E3.** Experimental schedule and dates of blood collection in patients 1 and 2.

| **Period of experiments** | **Patient 1**  **(Time points)** | **Patient 2**  **(Time points)** |
| --- | --- | --- |
| 05.02.19 - 12.04.19 | 07.02.2019 | 14.02.2019 |
| 28.05.19 - 04.07.19 | 29.05.2019 | 19.06.2019 |
| 05.09.19 - 21.10.19 | 13.09.2019 | 13.09.2019 |

**TABLE E4.** Clinical and demographic characteristics of patients 1 and 2.

|  |  |  |  |  |  |  |  |  |  |
| --- | --- | --- | --- | --- | --- | --- | --- | --- | --- |
|  | **Sex** | **Age** | **Symptoms related to birch pollen and apple** | | | **Symptoms of OAS to apple** | | | **Other symptoms** |
|  |  |  |  |  |  | **Time points** | | |  |
|  |  |  | **AD** | **Asthma** | **ARC** | **1** | **2** | **3** |  |
| **Patient 1** | female | 19 | no | yes | yes | yes | yes | yes | Asthma related to cat/dog allergens |
| **Patient 2** | female | 24 | no | no | yes | no | yes | yes | ARC related to cat/dog allergens |

**TABLE E5.** Molecular profiles of IgE sensitizations in patients 1 and 2.

|  | **rBet v 1** | **rCan f 1** | **rCan f 5** | **nCyn d 1** | **rFel d 1** | **rPhl p 1** | **rPru p 3** | **rVes v 5** |
| --- | --- | --- | --- | --- | --- | --- | --- | --- |
| **Patient 1** | 0.44 | 5.77 | 0 | 0 | 28.8 | 1.97 | 0 | 0 |
| **Patient 2** | 0.37 | 0 | 5.85 | 0.55 | 3.63 | 1.73 | 1.33 | 0.36 |

| **< 0.3 ISU-E** | Non-detectable |  |
| --- | --- | --- |
| **0.3-0.9 ISU-E** | Low reactivity |  |
| **1-14.9 ISU-E** | Moderate/high reactivity |  |
| **≥15 ISU-E** | Very high reactivity |  |

**TABLE E6.** Basophil activation results for patients 1 and 2.

| **Time points** | | **rBet v 1** | | | **rMal d 1** | | | **Anti-IgE** |
| --- | --- | --- | --- | --- | --- | --- | --- | --- |
|  |  | **1 ng/ml** | **10 ng/ml** | **100 ng/ml** | **1 ng/ml** | **10 ng/ml** | **100 ng/ml** |  |
| **Patient 1** | 1 | 0 | 1.41 | 17.87 | 1.97 | 1 | 6.59 | 46.57 |
|  | 2 | 0 | 2.04 | 11.26 | 13.76 | 10.56 | 9.06 | 69.76 |
|  | 3 | 2.33 | 6.95 | 0.25 | 0 | 8.35 | 16.35 | 7.69 |
| **Patient 2** | 1 | 0.04 | 0 | 2.47 | 0.1 | 1.52 | 0 | 23.45 |
|  | 2 | 27.58 | 79.28 | 63.58 | 56.28 | 9.38 | 21.28 | 88.48 |
|  | 3 | 0.33 | 0 | 2.46 | 8.2 | 4.13 | 4.12 | 44.68 |

| **< 5%** | Negative reactivity |  |
| --- | --- | --- |
| **5 – 10%** | Low reactivity |  |
| **10 – 20%** | Medium reactivity |  |
| **>20%** | High reactivity |  |

**TABLE E7.** Skin prick test results for patients 1 and 2.

| **Time points** | | **Negative control, mm^2^** | **Positive control histamine, mm^2^** | **Prick-to-prick test with apple, mm^2^** |
| --- | --- | --- | --- | --- |
| **Patient 1** | 1 | 0 | 150.4 | 210.6 |
|  | 2 | 0 | 146.3 | 308.2 |
|  | 3 | 0 | 144.8 | 280.2 |
| **Patient 2** | 1 | 0 | 115.6 | 35.6 |
|  | 2 | 0 | 117.6 | 110.1 |
|  | 3 | 0 | 118.1 | 150.5 |

**TABLE E8. Cumulative allergen-specific IgE (ISAC).**

| **Group 1** | | **Group 2** | |
| --- | --- | --- | --- |
| Patient № | Total IgE, ISU-E | Patient № | Total IgE, ISU-E |
| 1-1 | 58.13 | 2-1 | 28.14 |
| 1-2 | 182.76 | 2-2 | 28.21 |
| 1-3 | 453.34 | 2-3 | 107.04 |
| 1-4 | 220.84 | 2-4 | 29.32 |
| 1-5 | 500.06 | 2-5 | 12.49 |
| 1-6 | 48.41 | 2-6 | 22.65 |
| 1-7 | 30.23 | 2-7 | 21.08 |
| 1-8 | 109.99 | 2-8 | 71.18 |
| 1-9 | 32.98 | 2-9 | 94.83 |
| 1-10 | 18.56 | 2-10 | 465.48 |
| 1-11 | 722.12 | 2-11 | 89.56 |
| 1-12 | 174.72 | 2-12 | 30.01 |
| 1-13 | 11.06 |  | |

|  | Measurements of Patient 1 |
| --- | --- |
|  | Measurements of Patient 2 |

**SUPPLEMENTARY FIGURE LEGEND**

**FIGURE E1.** Summary of the study, results and interpretation.

**SUPPLEMENTARY REFERENCES**

E1 Asher MI, Keil U, Anderson HR, et al. International Study of Asthma and Allergies in Childhood (ISAAC): rationale and methods. *Eur Respir J*. 1995;**8**(3):483-491. doi:10.1183/09031936.95.08030483

E2 von Mutius E. Epidemiology of asthma: ISAAC--International Study of Asthma and Allergies in Childhood. *Pediatr Allergy Immunol*. 1996;**7**(9 Suppl):54-56. doi:10.1111/j.1399-3038.1996.tb00396.x

E3 Bousquet J, Heinzerling L, Bachert C, et al. Practical guide to skin prick tests in allergy to aeroallergens. *Allergy*. 2012;**67**(1):18-24. doi:10.1111/j.1398-9995.2011.02728.x

E4 Brożek JL, Bousquet J, Agache I, et al. Allergic Rhinitis and its Impact on Asthma (ARIA) guidelines-2016 revision. *J Allergy Clin Immunol*. 2017;**140**(4):950-958. doi:10.1016/j.jaci.2017.03.050

E5 Wollenberg A, Oranje A, Deleuran M, et al. ETFAD/EADV Eczema task force 2015 position paper on diagnosis and treatment of atopic dermatitis in adult and paediatric patients. *J Eur Acad Dermatol Venereol*. 2016;**30**(5):729-747. doi:10.1111/jdv.13599

E6 Skypala IJ, Calderon MA, Leeds AR, Emery P, Till SJ, Durham SR. Development and validation of a structured questionnaire for the diagnosis of oral allergy syndrome in subjects with seasonal allergic rhinitis during the UK birch pollen season. *Clin Exp Allergy*. 2011;**41**(7):1001-1011. doi:10.1111/j.1365-2222.2011.03759.x

E7 Position paper: Allergen standardization and skin tests. The European Academy of Allergology and Clinical Immunology. *Allergy*. 1993;**48**(14 Suppl):48-82.

E8 Garib V, Ben-Ali M, Kundi M, et al. Profound differences in IgE and IgG recognition of micro-arrayed allergens in hyper-IgE syndromes. *Allergy*. 2022; **77**(6):1761-1771. doi: 10.1111/all.15143. Epub 2021 Nov 2

E9 Curin M, Huang HJ, Garmatiuk T, et al. IgE Epitopes of the House Dust Mite Allergen Der p 7 Are Mainly Discontinuous and Conformational. *Front Immunol*. 2021;**12**:687294. Published 2021 Jun 15. doi:10.3389/fimmu.2021.687294

E10 Matthes A, Schmitz-Eiberger M. Apple (Malus domestica L. Borkh.) allergen Mal d 1: effect of cultivar, cultivation system, and storage conditions. *J Agric Food Chem*. 2009;**57**(22):10548-10553. doi:10.1021/jf901938q

E11 Vlieg-Boerstra BJ, van de Weg WE, van der Heide S, et al. Identification of low allergenic apple cultivars using skin prick tests and oral food challenges. *Allergy*. 2011;66(4):491-498. doi:10.1111/j.1398-9995.2010.02499.x
