# Supplementary material for: Allergic sensitization to Mal d 1 without detectable specific serum IgE
Source: Pediatr Allergy Immunol. 2022 Dec 7;33(12):e13891. doi: 10.1111/pai.13891 (PMC10107674; doi:10.1111/pai.13891)

Study Population

25 Bet v 1 allergic patients

GROUP 1	GROUP 2
**With OAS to apple**	**Without OAS to apple**
N=13	N=12
Age, years M±SD 29.15±12.44	Age, years M±SD 36.33±14.9
9 males, 4 females	7 males, 5 females

Testing

1 2 3 **rBet v 1 rMal d 1 sIgE**

1 2 3 **rBet v 1 rMal d 1 BAT**

1 2 3 **SPT with apple**

Birch pollen season

Experimental time points: 1, 2, 3

Key finding

2 of 25 patients

	Bet v 1	Mal d 1
**sIgE**	positive	**negative**
**BAT**	positive	positive
**SPT**	positive	positive

Explanation

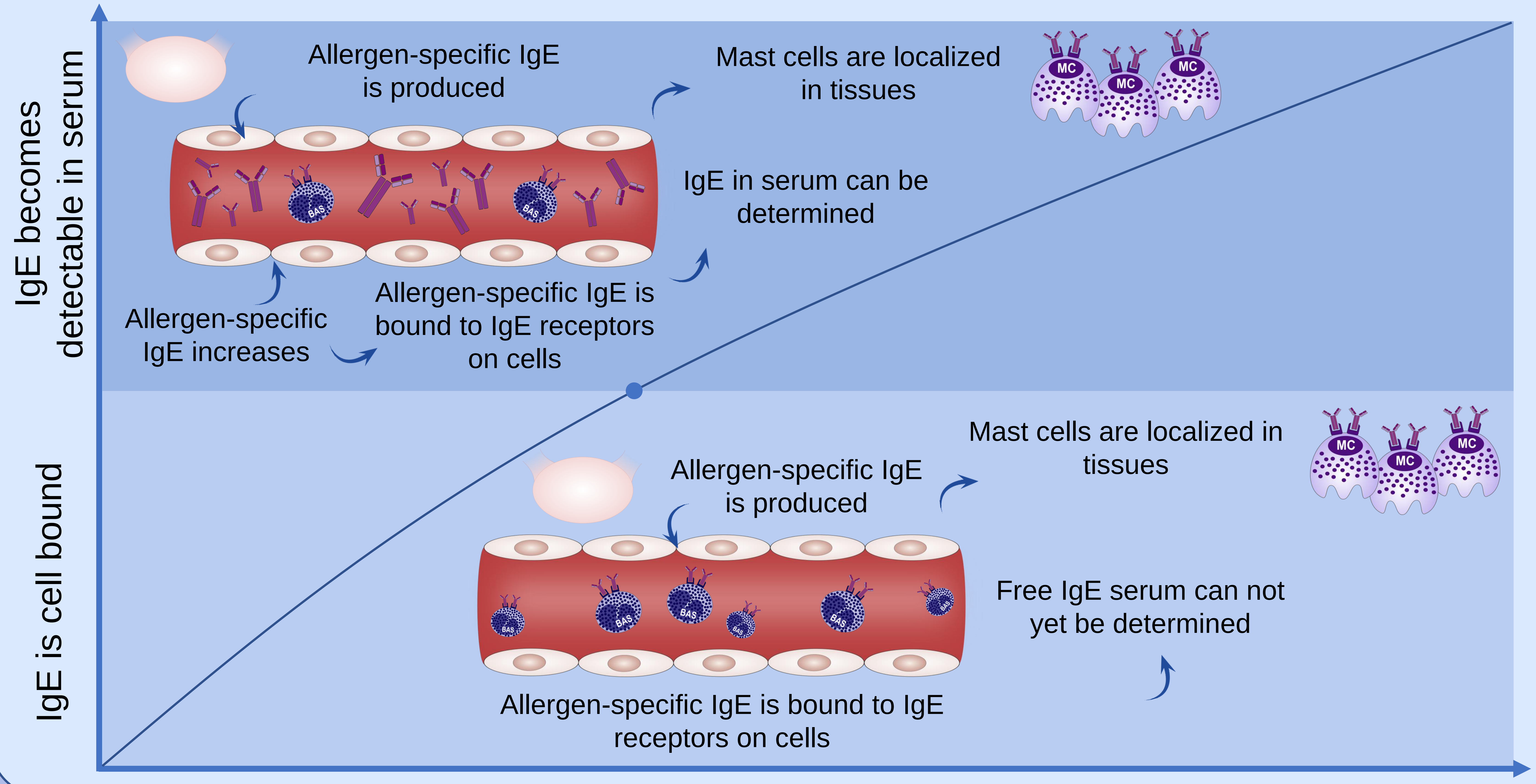

Supplement: Supplementary file 2 — FigureS1 [file PAI-33-0-s002.pdf]
